# Osteoporosis and Jaw Abnormalities in Panoramic Radiography of Chronic Liver Failure Patients

**DOI:** 10.1155/2018/4280312

**Published:** 2018-08-26

**Authors:** Janan Ghapanchi, Maryam Zahed, Abdolaziz Haghnegahdar, Niloofar Niakan, Azita Sadeghzadeh

**Affiliations:** ^1^Department of Oral and Maxillofacial Medicine, School of Dentistry, Shiraz University of Medical Sciences, Shiraz, Iran; ^2^Oral and Dental Disease Research Center, Department of Oral and Maxillofacial Medicine, School of Dentistry, Shiraz University of Medical Sciences, Shiraz, Iran; ^3^Department of Oral and Maxillofacial Radiology, School of Dentistry, Shiraz University of Medical Sciences, Shiraz, Iran; ^4^Student Research Committee, School of Dentistry, Shiraz University of Medical Sciences, Shiraz, Iran

## Abstract

**Introduction:**

Patients with chronic liver failure (CLF) are faced with many complications, because this organ is involved in various metabolic activities. Hepatic osteodystrophy is one of the major health issues encountered by this group of patients. The current study evaluated osteoporosis and bone changes in oral panoramic radiographies of cirrhotic patients.

**Materials and Methods:**

In this study, 138 panoramic views of CLF patients, candidates for liver transplant (65 females, 73 males, aged 19-68 years) referred to Shiraz University Dental Clinic (Shiraz, Iran) for pretransplant oral examination, were evaluated. Also 138 healthy individuals (69 females, 69 males, aged 18-70 years) referred to the same clinic were examined. Abnormalities such as osteoporosis, pathologic radiolucencies, pathologic calcifications, tonsilloliths, condylar degeneration, and other findings in the alveolar bones were recorded.

**Results:**

Osteoporosis was a common finding in CLF patients (*p*<0.001), and the probability of detecting low bone density in the panoramic view was 20.37 times higher among patients than healthy subjects. The probability of detecting pathologic jaw radiolucencies was 8.92 times higher in the case group than in the controls (*p*<0.001). Other bone abnormalities such as condylar degeneration and idiopathic osteosclerosis were also more prevalent in CLF patients compared to healthy subjects (*p*<0.001).

**Conclusion:**

Cirrhotic patients are prone to osteoporosis of the alveolar bones. Also, pathologic jaw radiolucencies as a result of oral infections are more prevalent in this group of patients. Routine oral panoramic views are acceptable and cost-effective radiographies for use in detecting such abnormalities in the alveolar bones as well as overall dental health. These findings also support the importance of dental health examinations prior to liver transplantation to reduce the risk of organ rejection.

## 1. Introduction

Liver disorders are one of the main causes of morbidity and mortality worldwide. With over one million deaths in 2010, cirrhosis of the liver is known as a major health burden [1].

Hepatic osteodystrophy is known as osteoporosis and osteomalacia in patients suffering from liver cirrhosis [2]. Bone density in chronic liver failure (CLF) patients has been previously evaluated, and all investigations have shown a decrease in bone density levels [3–5]. Liver dysfunction is known as the second main cause of osteoporosis or osteopenia [2]. It is reported that the estimation of bone density alteration in cirrhotic patients is 12% to 55% [4–7]. As a rule, low bone mass or density may act as significant risk factors for fracture. The rate of fracture in CLF patients is reported to be 5% to 20% and highly related to age and stage of disease [2].

The mechanism behind this bone abnormality is related to the distinct characteristic of the cause of liver disease. Nevertheless, the liver is involved in many metabolic activities; therefore, general factors such as vitamin D and calcium alterations and the resultant secondary hyperparathyroidism, vitamin K deficiency, hormonal dysfunction, cytokines, and deficiency of insulin-like growth factor 1 (IGF-1) all affect bone metabolism [2].

The number of patients with chronic liver failure (CLF) referred to dental health centers is increasing every year [8–10]. These cases must be persistently examined by a dentist before going through liver transplant procedures [8, 11]. Oral infections are considered a major cause of treatment failure in liver transplant recipients [10]. Besides the role of oral infections in the prognosis of CLF, oral lesions can be regarded as a diagnostic tool in the evaluation of the general health of the patient [5, 12]. Moreover, CLF, like many other systemic diseases (hematologic, endocrine, and metabolic disorders), can affect both the hard and soft tissues of the oral cavity [4, 12, 13].

All CLF cases undergo panoramic radiography prior to transplant in order to detect any source of infection in the oral cavity [14]. This radiography is inexpensive, extensively used, and easily performed and has a relatively low dose of radiation [15]. Furthermore, its use in evaluating bone density levels has been previously recognized [16, 17]. To the best of the authors' knowledge, however, there is no information about panoramic radiographies and low bone density in CLF cases. Therefore, this study is designed to assess osteoporosis and other radiographic changes in the panoramic views of CLF patients compared with normal individuals to better understand the hard tissue changes manifested in the alveolar bones of these patients.

## 2. Patients and Methods

### 2.1. Study Group

This analytical cross-sectional study was conducted on 138 panoramic views of CLF patients who were candidates for liver transplant (65 females, 73 males, aged 19-68 years) and 138 healthy individuals (69 females, 69 males, aged 18-70 years). All cases were referred to Shiraz University Dental Clinic (Shiraz Dental School, Shiraz, Iran) for a pretransplant oral examination. A control group was also randomly selected from healthy individuals who attended the same clinic for routine dental care between March 2015 and August 2017. After obtaining written informed consent from each participant, dentate patients with acceptable quality panoramic radiographs were entered into the study. Cases with a history of anemia, head and neck radiotherapy, diabetes mellitus, corticosteroid consumption, smoking and narcotic use, rheumatic disease, pregnancy, tonsillectomy, or metabolic bone disease (thyroid disorders, hyperparathyroidism, Addison's and Cushing disease) were excluded from the study. Demographic data collected included gender, age, and duration of disease.

### 2.2. *Imaging Procedures*

Panoramic views were prepared using a Planmeca XC Proline panoramic machine (Helsinki, Finland). Exposure factors were adjusted according to the size and age of the patient (57-85 kVp, 10 mA), using Agfa PSP receptors (Germany). The images were observed on a Barco monitor (China) in a semi-darkened room. All radiographs were evaluated by an oral and maxillofacial radiologist and an oral medicine specialist to achieve a capital value of agreement. Osteoporosis and other abnormalities such as condylar degeneration, pathologic radiolucency, calcifications, tonsilloliths, and other findings in the jawbone and temporomandibular joint were recorded.

**Figure 1:**
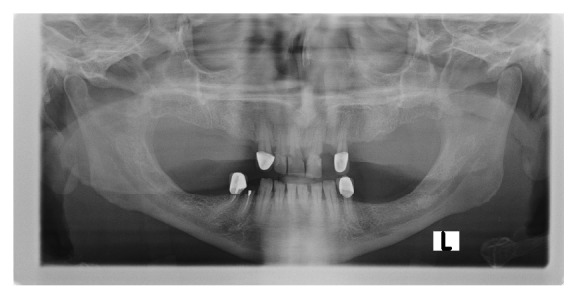
Osteoporosis in chronic liver failure patients seen as rarefying of the bone and thinning of cortex.

In the panoramic radiography, osteoporosis was defined as low bone mass and micro-architectural changes of bone tissue, rarefying of the bone, thinning of the cortex, and loss of the lamina dura (Figure 1) [18].

**Figure 2:**
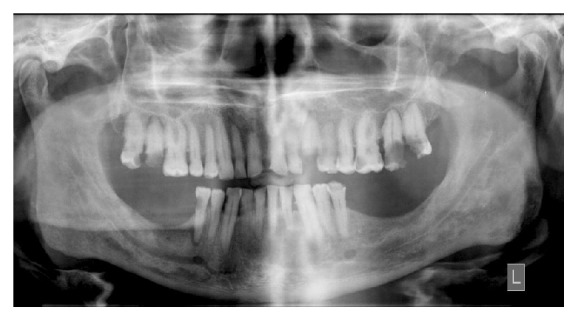
Multiple apical radiolucencies seen in the mandibular and maxillary dentition of a chronic liver failure patient.

Panoramic radiographs were inspected for the presence of radiolucent jaw lesions of both odontogenic and nonodontogenic origin with different etiologies (reactive, benign, or malignant) (Figure 2) [10].

**Figure 3:**
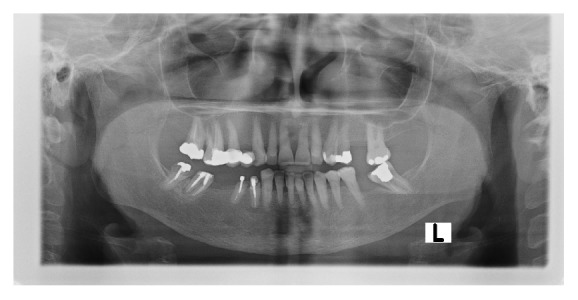
Condylar degeneration in the left mandibular condyle of a chronic liver failure patient.

Temporomandibular joint disorders (TMD) were considered as degenerative changes of the articular bone and presence of osteophytes on the panoramic radiographies [19](Figure 3).

Idiopathic osteosclerosis (IO) due to increased bone production in the jaw was found as a round, elliptical, or irregular radiopaque area [20].

**Figure 4:**
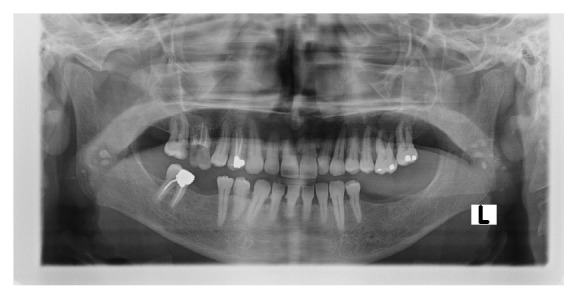
Bilateral tonsilloliths presented as radiopaque lesions superimposed on the mandibular ramus in a chronic liver failure patient.

Tonsilloliths usually appear as numerous small and ill-defined radiopacities located on the ramus and usually superimposed over the border of the tongue shadow (Figure 4) [15, 21].

### 2.3. *Ethical Considerations*

The research followed the tenets of the Declaration of Helsinki. Written informed consent was obtained from all patients who participated in the study. All information about individuals was coded and kept confidential. This study was approved by the Ethics Committee of Shiraz University of Medical Sciences.

### 2.4. *Statistical Analysis*

The data was collected and analyzed using SPSS software (version 18; SPSS Inc., Chicago, IL, USA). The chi-square and odds ratio tests (95% confidence interval) were performed to compare the findings between the case and control groups. A* p* value < 0.05 was accepted as significant.

## 3. Results

A total of 138 panoramic views of patients with CLF (65 females, 73 males, aged 19-68 years) and 138 healthy subjects (69 females, 69 males, aged 18-70 years) were reviewed in the Oral and Maxillofacial Radiology Clinic of the University of Shiraz, Iran. The duration of the liver disease in the case group varied between 2 and 223 months with a medium duration of 67.3 ± 52.8 months.

Osteoporosis was a common finding in CLF patients (*p*<0.001), and the probability of detecting such lesions in end-stage liver disease was 20.37 times higher in patients than in control subjects (95% CI=6.14-67.59) (Table 1).

Pathologic jaw radiolucency was more prevalent in CLF patients than in healthy subjects (*p*<0.001). In comparison, the probability of detecting such lesions in the case group was 8.92 times higher than controls (95% CI=2.01-39.58) (Table 1).

Based on the results of the chi-square test, the data revealed a significant difference between cases and controls in the presence of condylar degeneration (*p*<0.001). The chance of condylar involvement was 4.2 times higher in the case group than control (95% CI=2.40-7.35) (Table 1).

There was no significant relation between the groups in presence of tonsilloliths (*p*=0.198, OR=0.42) (Table 1).

Statistical analysis demonstrated a significant correlation between the detection of idiopathic osteosclerosis (IO) and liver disease (*p*<0.001). The chance of the presence of IO was 12.73 higher in the case group than in the controls (95% CI=4.40-36.83) (Table 1).

## 4. Discussion

The organs in the human body have an interdependent relationship, such that any pathologic condition in one element can alter the health status in other body parts. One of the main complications for patients with medical problems is bone manifestations, especially those affecting the jaws. The main finding of the current study is that osteoporosis is commonly detected in the panoramic radiographies of liver cirrhosis patients. Moreover, pathologic radiolucencies that are mostly the result of dental infections and poor oral health are more prevalent in this group of patients.

Digital panoramic radiographs are taken routinely for the diagnosis and management of individuals referred to orodental clinics. Generally, intraoral radiographs are more popular for the detection of dental-related disorders, but they are not suitable for evaluating bony changes [17]. Advanced radiography such as cone beam computed tomography (CBCT) is thought to be superior to panoramic views in detecting precise changes in bone structure due to the lack of superimposition and distortion. On the other hand, this radiography exposes the patient to more radiation and is more expensive [22]. Furthermore, it is mentioned in the literature that panoramic radiography is as good as CBCT in detecting anatomical structures [23]. Bone resorption and calcifications may easily be detected in this radiography, and panoramic indices such as thickness of the mandibular cortex can be used to show osteoporosis [17]. Dual energy X-ray absorptiometry (DXA) is widely used to evaluate bone mineral density; however, it is not routinely recommended because it is not cost beneficial [2, 17]. Moreover, considering the fact that the accuracy of densitometry is reduced in patients with ascites because of the superimposition of fluid both at the lumbar spine and the proximal femur, new modalities are required as diagnostic options in liver failure patients [2].

In the current study, panoramic radiographs revealed osteoporosis in approximately one-third of CLF patients. In agreement with this, previous studies have reported the inferior mandibular cortex density to be reduced in panoramic views of CLF patients compared with normal individuals [14]. This result is also in accordance with studies that have shown a decrease in bone density as high as 68% in liver transplant candidates [5]. In 2016, a study was carried out to evaluate osteopenia and osteoporosis in patients with various chronic liver diseases. The researchers concluded that low bone mineral density is highly prevalent in this group of patients, and vitamin D levels and severity of liver disease are correlated with low BMD [4]. The pathogenic mechanism underlying this reduction is hypothesized in the literature to be a decrease in the proliferative capacity of osteoblasts with increasing bilirubin levels, which is dose dependent [4, 5, 24, 25]. It is noteworthy that vitamin D3 is hydroxylated in the liver. The metabolism of calcium and vitamin D is impaired in liver cirrhosis with the resultant parathyroid hormone (PTH) disturbance [2]. Gatta et al. suggest densitometry screening for osteoporosis for all patients with advanced chronic liver disease. Moreover, bone mineral density is reduced rapidly in the first six months after transplantation; thus, bone density values are very important prognostic factors at the time of transplantation [7]. Early diagnosis and treatment of this disorder are of high importance due to its serious complications. Eliminating risk factors, such as ceasing tobacco and alcohol consumption; reducing caffeine ingestion; exercise; supplementation with calcium and vitamin D; and limiting the intake of drugs such as loop diuretics, corticosteroids, and cholestyramine constitute the first line of therapy [7].

Another finding suggestive of osteoporosis in the panoramic radiographs of the present study was condylar degeneration, which was significantly more common in the case group. As reported in previous studies, the mandibular condyle can be a biomarker in panoramic views of patients for the early detection of osteoporosis [26]. The fibrocartilage of the temporomandibular joint is located above the bone of the mandibular condyles; this makes the bone very vulnerable to inflammatory damage due to systemic disease and a valuable model for studying arthritic bony changes [27]. Also it is mentioned in the literature that psychological stress is correlated with temporomandibular disorders (TMDs) [28]. Compromised systemic patients are faced with many psychological and emotional stresses when dealing with their illness. Osteoporosis in conjunction with psychological stress can be the main cause of condylar degeneration in this group.

Panoramic radiographs also identified pathologic radiolucencies as a cause of apical periodontitis and other pathologic causes (nonodontogenic) in almost 12% of the evaluated cases in this study. This finding was significantly more common in liver cirrhosis patients than in healthy individuals. PTH disturbances and secondary hyperparathyroidism can cause radiolucent jaw lesions referred to as “Brown Tumors” [29]. Although vitamin D levels are decreased in liver cirrhosis patients, secondary hyperparathyroidism is found to be relatively uncommon [30]. Furthermore, apical radiolucency is most often the result of necrotic dental pulp which develops when oral bacteria reach the dental pulp through large and deep caries lesions of the teeth [10, 31]. Therefore the causes of radiolucent lesions are mainly tooth-related, as has been seen in other studies examining the presence of tooth apical radiolucencies in liver cirrhosis patients [10, 11]. In 2016, researchers found this lesion in 46% of their cases. They also revealed that patients with periapical radiolucencies had a higher prevalence of cirrhosis-related complications such as ascites, hepatic encephalopathy, and/or variceal bleeding, although neither cirrhosis etiology (alcoholic versus nonalcoholic) nor severity (model of end-stage liver disease (MELD) score) was a predictor for the prevalence of jaw radiolucencies. [10]. Other studies support this finding, showing that the etiology of chronic liver disease and the severity of the disease are associated with oral health status; the higher the MELD score is, the more undesirable the dental health condition is [11]. Moreover, the oral health of candidates of solid organ transplants is an important matter due to the permanent immunosuppression they face after transplantation [12]. In the United States, a study was carried out in 294 transplant centers, of which 38% showed a spread of infection after transplantation due to a dental source. At least one case of posttransplantation sepsis with a possible odontogenic source was recorded in 27% of treatment centers [32]. These lesions can precipitate systemic inflammation activation in both healthy and compromised patients [31]. Idiopathic sclerosis was also more prevalent in the case group. This lesion must be distinguished from remnants of a past infectious lesion [20]. Thus, the need for a more thorough oral examination in order to eliminate any source of dental infection or a workup to detect secondary hyperparathyroidism is also supported in the cases of this study.

Shiraz University of Medical Sciences is the main center for referral of CLF patients in Iran. Therefore, the cases were easy to access, and a good number of patients were evaluated. However, further evaluations on a larger number of cases are suggested.

## 5. Conclusion

Panoramic views are relevant radiographies for the detection of osteoporosis in the alveolar bones of liver cirrhotic patients. This can reduce the need for expensive and invasive workups to detect low bone density in these compromised cases. Pathologic alveolar radiolucencies as a result of tooth infection or secondary hyperparathyroidism are also more prevalent in CLF patients. Panoramic radiographs are feasible and cost-effective instruments that help with the overall health of the patient by detecting such lesions. Radiolucent bone pathologies need further assessment, because they can represent sources of infection after liver transplantation. The study findings also reveal that cirrhotic patients are more likely to have poorer oral health than healthy individuals; thus, the current study supports a thorough dental screening and close monitoring for dental infections in candidates of liver transplantation.

## Figures and Tables

**Table 1:** Comparison of oral panoramic radiographic findings in chronic liver failure patients with those of the healthy control group.

Radiographic findings		Group				*p* value*∗∗*	OR (95% CI)*∗∗∗*
		Case		Control			
		No.^*∗*^	percentage	No.	percentage		
Osteoporosis	Negative	195	68.8%	135	97.8%	<0.001	20.37 (6.14- 67.59)
	Positive	43	31.2%	3	2.2%		

Pathologic Radiolucency	Negative	122	88.4%	136	98.6%	0.001	8.92(2.01- 39.58)
	Positive	16	11.6%	2	1.4%		

Condylar degeneration	Negative	75	54.3%	115	83.3%	<0.001	4.2 (2.40-7.35)
	Positive	63	45.7%	23	16.7%		

Idiopathic Osteosclerosis	Negative	100	72.5%	134	97.1%	<0.001	12.73 (4.40-36.83)
	Positive	38	27.5	4	2.9%		

Tonsilloliths	Negative	135	97/8%	131	94/9%	0.198	0.42(0.11-1.64)
	Positive	3	2.2%	7	5.1%		

Total		138		138		-	-

*∗*Number; *∗∗*chi-square test; *∗∗∗*odds ratio (95% confidence interval)

## Data Availability

Other radiographies used to support the findings of this study are available upon request from the corresponding author.

## References

[1] Smith B. W., Adams L. A. (2011). Non-alcoholic fatty liver disease. *Critical Reviews in Clinical Laboratory Sciences*.

[2] Handzlik-Orlik G., Holecki M., Wilczyński K., Duława J. (2016). Osteoporosis in liver disease: pathogenesis and management. *Therapeutic Advances in Endocrinology and Metabolism*.

[3] Corey R. L., Whitaker M. D., Crowell M. D., Keddis M. T., Aqel B., Balan V. (2014). Vitamin D deficiency, parathyroid hormone levels, and bone disease among patients with end‐stage liver disease and normal serum creatinine awaiting liver transplantation. *Clinical Transplantation*.

[4] Karoli Y., Karoli R., Fatima J., Manhar M. (2016). Study of hepatic osteodystrophy in patients with chronic liver disease. *Journal of Clinical and Diagnostic Research*.

[5] Patel N., Muñoz S. J. (2015). Bone disease in cirrhosis. *Clinical Liver Disease*.

[6] Eastell R., Dickson E. R., Hodgson S. F. (1991). Rates of vertebral bone loss before and after liver transplantation in women with primary biliary cirrhosis. *Hepatology*.

[7] Gatta A., Verardo A., Di Pascoli M., Giannini S., Bolognesi M. (2014). Hepatic osteodystrophy. *Clinical Cases in Mineral and Bone Metabolism*.

[8] Guggenheimer J., Eghtesad B., Close J. M., Shay C., Fung J. J. (2007). Dental health status of liver transplant candidates. *Liver Transplantation*.

[9] Helenius-Hietala J., Ruokonen H., Grönroos L. (2013). Self-reported oral symptoms and signs in liver transplant recipients and a control population. *Liver Transplantation : Official Publication of the American Association for the Study of Liver Diseases and the International Liver Transplantation Society*.

[10] Gronkjær L., Holmstrup P., Schou S., Schwartz K., Kongstad J., Jepsen P. (2016). Presence and consequence of tooth periapical radiolucency in patients with cirrhosis. *Hepatic Medicine: Evidence and Research*.

[11] Helenius-Hietala J., Meurman J. H., Höckerstedt K., Lindqvist C., Isoniemi H. (2012). Effect of the aetiology and severity of liver disease on oral health and dental treatment prior to transplantation. *Transplant International*.

[12] Helenius-Hietala J., Ruokonen H., Grönroos L. (2014). Oral mucosal health in liver transplant recipients and controls. *Liver Transplantation*.

[13] Ghapanchi J., Rezaee M., Kamali F., Lavaee F., Shakib E. (2014). Prevalence of oral and craniofacial manifestations of hematological dyscrasias at Shiraz Nemazee Hospital. *Middle East Journal of Cancer*.

[14] Ghapanchi J., Haghnegahdar A. A., Faghih M. (2017). Evaluation of mandibular inferior cortex changes in patients candidate for liver and kidney transplantation using panoramic view. *Journal of Nephropathology*.

[15] Sutter W., Berger S., Meier M., Kropp A., Kielbassa A. M., Turhani D. (2018). Cross-sectional study on the prevalence of carotid artery calcifications, tonsilloliths, calcified submandibular lymph nodes, sialoliths of the submandibular gland, and idiopathic osteosclerosis using digital panoramic radiography in a lower Austrian subpopulation. *Quintessence International*.

[16] Calciolari E., Donos N., Park J. C., Petrie A., Mardas N. (2015). Panoramic measures for oral bone mass in detecting osteoporosis: A systematic review and meta-analysis. *Journal of Dental Research*.

[17] Kim O.-S., Shin M.-H., Song I.-H. (2016). Digital panoramic radiographs are useful for diagnosis of osteoporosis in Korean postmenopausal women. *Gerodontology*.

[18] Taguchi A. (2009). Panoramic radiographs for identifying individuals with undetected osteoporosis. *Japanese Dental Science Review*.

[19] Bäck K., Ahlqwist M., Hakeberg M., Dahlström L. (2017). Occurrence of signs of osteoarthritis/arthrosis in the temporomandibular joint on panoramic radiographs in Swedish women. *Community Dentistry and Oral Epidemiology*.

[20] Fuentes R., Arias A., Astete N., Farfán C., Garay I., Dias F. (2018). Prevalence and morphometric analysis of idiopathic osteosclerosis in a Chilean population. *Folia Morphologica*.

[21] Takahashi A., Sugawara C., Kudoh T. (2017). Prevalence and imaging characteristics of palatine tonsilloliths evaluated on 2244 pairs of panoramic radiographs and CT images. *Clinical Oral Investigations*.

[22] Lim L., Padilla R., Reside G., Tyndall D. (2017). Radiographic Features And Differential Diagnoses Of Pathology: Comparing Panoramic Radiographs and Cbct. *Oral Surgery, Oral Medicine, Oral Pathology and Oral Radiology*.

[23] Neves F. S., Nascimento M. C. C., Oliveira M. L., Almeida S. M., Bóscolo F. N. (2013). Comparative analysis of mandibular anatomical variations between panoramic radiography and cone beam computed tomography. *Oral and Maxillofacial Surgery*.

[24] Liu Z., Han T., Werner H., Rosen C. J., Schaffler M. B., Yakar S. (2018). Reduced Serum IGF‐1 Associated With Hepatic Osteodystrophy Is a Main Determinant of Low Cortical but Not Trabecular Bone Mass. *Journal of Bone and Mineral Research*.

[25] Culafić D., Djonic D., Culafic-Vojinovic V. (2014). Evidence of degraded BMD and geometry at the proximal femora in male patients with alcoholic liver cirrhosis. *Osteoporosis International*.

[26] Kosugi K., Yonezu H., Kawashima S., Honda K., Arai Y., Shibahara T. (2013). A longitudinal study of the effect of experimental osteoporosis on bone trabecular structure in the rat mandibular condyle. *Cranio: Journal of Craniomandibular Practice*.

[27] Cevidanes L. H. S., Walker D., Schilling J. (2014). 3D osteoarthritic changes in TMJ condylar morphology correlates with specific systemic and local biomarkers of disease. *Osteoarthritis and Cartilage*.

[28] Lv X., Li Q., Wu S., Sun J., Zhang M., Chen Y.-J. (2012). Psychological stress alters the ultrastructure and increases IL-1^2^ and TNF-± in mandibular condylar cartilage. *Brazilian Journal of Medical and Biological Research*.

[29] dos Santos B., Koth V. S., Figueiredo M. A., Salum F. G., Cherubini K. (2018). Brown tumor of the jaws as a manifestation of tertiary hyperparathyroidism: A literature review and case report. *Special Care in Dentistry*.

[30] Miroliaee A., Nasiri-Toosi M., Khalilzadeh O., Esteghamati A., Abdollahi A., Mazloumi M. (2010). Disturbances of parathyroid hormone–vitamin D axis in non-cholestatic chronic liver disease: a cross-sectional study. *Hepatology International*.

[31] Gomes M. S., Blattner T. C., Sant'Ana Filho M., Grecca F. S., Hugo F. N., Fouad A. F. (2013). Can apical periodontitis modify systemic levels of inflammatory markers? A systematic review and meta-analysis. *Journal of endodontics*.

[32] Guggenheimer J., Mayher D., Eghtesad B. (2005). A survey of dental care protocols among US organ transplant centers. *Clinical Transplantation*.

